# Overview of approved CAR-T products and utility in clinical practice

**DOI:** 10.46989/001c.124277

**Published:** 2024-10-23

**Authors:** Shakthi T Bhaskar, Bhagirathbhai Dholaria, Bipin N Savani, Salyka Sengsayadeth, Olalekan Oluwole

**Affiliations:** 1 Medicine, Hmatology and Oncology Vanderbilt University Medical Center; 2 Vanderbilt University Medical Center

**Keywords:** Chimeric antigen receptor, approved, T Cell, approved products

## Introduction

Since the first chimeric antigen receptor (CAR) T-cell product received FDA approval in 2017, studies have been completed that have led to approvals of CAR T-cells to treat a variety of malignancies (Table 1). CAR T-cell therapy is associated with side effects different from conventional chemotherapy. These include immediate side effects such as cytokine release syndrome (CRS), immune effector cell-associated neurotoxicity (ICANS), and long-term side effects such as hypogammaglobulinemia and persistent cytopenias. Irrespective of side effects, CAR T-cells have expanded the therapies available for relapsed and refractory hematologic malignancies and significantly improved patient outcomes. Below, we review the currently commercially available CAR T-cell products and their indications as of 2023.

**Table 1.** Overview of approved CAR-T products and utility in clinical practice.

**Product Name, Costimulatory Domain**	**Target**	**Disease Approval**	**Approval Year**	**Relevant Studies**	**Line of Therapy**	**Other**
Axicabtagene ciloleucel (axi-cel, Yescarta ^©^), CD28	CD19	Large B-cell lymphoma	April 2022 October 2017	ZUMA-7 (NCT03391466) ZUMA-1 (NCT02348216)	Second line if primary refractory or relapsed within 12 months of first-line treatment R/R after 2 or more lines of treatment*	*Included diffuse large B-cell, primary mediastinal, high-grade B-cell, and transformed follicular
		Follicular lymphoma	March 2021	ZUMA-5 (NCT03105336)	R/R after 2 or more lines of treatment	
Tisagenlecleucel (tisa-cel, Kymriah^©^), 4-1BB	CD19	B-cell precursor acute lymphoblastic leukemia	August 2017	ELIANA (NCT02435849)	Refractory or relapsed after 2 or more lines of treatment	
		Large B-cell lymphoma	May 2018	JULIET (NCT02445248)	R/R after 2 or more lines of treatment**	**Included diffuse large B-cell, high-grade B-cell, and transformed follicular
		Follicular lymphoma	May 2022	ELARA (NCT03568461)	R/R after 2 or more lines of treatment	
Brexucabtagene autoleucel (brexu-cel, Tecartus^©^), CD28	CD19	Mantle cell lymphoma	July 2020	ZUMA-2 (NCT02601313)	Relapsed or refractory	
		B-cell precursor acute lymphoblastic leukemia	October 2021	ZUMA-3 (NCT02614066)	Relapsed or refractory	
Lisocabtagene maraleucel (liso-cel, Breyanzi^©^), 4-1BB	CD19	Large B-cell lymphoma***	June 2022 February 2021	TRANSFORM (NCT02631044) TRANSCEND NHL-001 (NCT02631044)	Second line if primary refractory or relapsed within 12 months of first-line treatment Primary refractory or relapsed after first-line treatment and not autologous stem cell transplant candidate R/R after 2 or more lines of treatment	***Included diffuse large B-cell, transformed indolent lymphoma, high grade B-cell, primary mediastinal, and follicular lymphoma grade 3B
Idecabtagene vicleucel (ide-cel, Abecma^©^), 4-1BB	B-cell maturation antigen (BCMA)	Multiple myeloma	March 2021	KarMMa (NCT03361748) KarMMa-3 (NCT03651128)	R/R after four or more prior lines including immunomodulatory agent, proteasome inhibitor, and anti-CD38 monoclonal antibody	
Ciltacabtagene autoleucel (cilta-cel, Carvykti^©^), 4-1BB	BCMA	Multiple myeloma	February 2022	CARTITUDE-1 (NCT03548207) CARTITUDE-4 (NCT04181827)	R/R after four or more prior lines including immunomodulatory agent, proteasome inhibitor, and anti-CD38 monoclonal antibody	

## Axicabtagene ciloleucel

Axicabtagene ciloleucel (axi-cel, Yescarta©) is a CD19 CAR product generated from autologous lymphocytes. It is manufactured by use of a gamma retroviral vector followed by expansion and product preparation and release testing. This CAR has a CD28 costimulatory domain, in contrast with other CAR T-cell products (see below).

Axi-cel was first approved in 2017 for treatment of relapsed/refractory (R/R) large B-cell lymphomas (LBCL) based on the results of the ZUMA-1 study (NCT02348216).^1^ In this heavily pretreated population of 111 patients, axi-cel showed an objective response rate (ORR) of 82% complete response (CR) rate of 54%. Overall survival (OS) at 18 months was 52%. The trial included 77 (76%) patients with diffuse large B-cell lymphoma (DLBCL), 8 (8%) patients with primary mediastinal B-cell lymphoma (PMBCL), and 16 (16%) patients with transformed follicular lymphoma (tFL). The FDA approval based on this study includes patients with DLBCL, PMBCL, tFL, and high-grade B-cell lymphoma (HGBCL).

The subsequent ZUMA-7 (NCT03391466) study compared the use of axi-cel to autologous stem cell transplant (ASCT) in patients with primary refractory DLBCL or with DLBCL that had relapsed within 12 months of initial treatment.^2^ This study resulted in superior median event free survival (EFS) of 8.3 months in the axi-cel group compared to 2.0 months in the standard of care group. Patients who received axi-cel had a significantly higher ORR of 83% and CR rate of 65% compared with 50% and 32% respectively in the standard of care arm. Longer follow up showed an OS benefit amongst the patients treated with axi-cel, with median OS in the axi-cel group not reached compared to 31.1 months in the standard of care group.^3^

Axi-cel was also studied in indolent non-Hodgkin lymphomas (NHL) in the ZUMA-5 study (NCT03105336) which included 153 patients with R/R follicular lymphoma (FL) and marginal zone lymphoma (MZL).^4^ In this non-randomized study, axi-cel showed a favorable ORR of greater than 90% in FL cohort. Based on this, the FDA granted accelerated approval to axi-cel for patients with R/R follicular lymphoma pending confirmatory results from ongoing studies (NCT05605899). Approval for axi-cel in MZL has not been granted at this time.

Additional studies are underway evaluating axi-cel for other indications. These include the ZUMA-23 study (NCT05605899) which is evaluating axi-cel as first-line treatment for patients with LBCL compared with standard of care chemoimmunotherapy. ZUMA-22 (NCT05371093) is a phase 3 trial evaluating axi-cel compared with standard of care treatment for patients with R/R FL (as follow up to the ZUMA-5 study).

## Tisagenlecleucel

Tisagenlecleucel (tisa-cel, Kymriah©) is a CD19 CAR product generated from autologous lymphocytes. Its manufacturing process involves transduction with a lentiviral vector. The CAR is accompanied by the 4-1BB costimulatory domain, which has been shown to lead to improved expansion and persistence of CAR T-cells in vivo.

Tisa-cel was first approved in 2017 for treatment of patients up to age 25 with R/R B-cell precursor acute lymphoblastic leukemia (B-ALL) based on the ELIANA study (NCT02435849).^5^ In this study treatment with tisa-cel resulted in a 12-month OS of 76% in the 75 patients who were included. Tisa-cel was later approved for use in R/R large B-cell lymphomas, including DLBCL, HGBCL, and tFL, based on the JULIET study (NCT02445248) which resulted in a 12-month relapse free survival of 65%.^6^ Finally, tisa-cel has also been approved for use in R/R FL based on the ELARA study (NCT03568461) which reported a CR rate of 69.1% and, after longer follow up, reported a 24-month OS of 87.7%.^7^

Of note, the BELINDA trial (NCT03570892) evaluated the use of tisa-cel compared to ASCT in patients with large B-cell lymphoma whose disease was either primary refractory or relapsed within 12 months of receiving first-line chemoimmunotherapy.^8^ This study demonstrated no significant difference in event-free survival between the two arms and tisa-cel is not approved as second-line therapy. The negative finding in this study is thought to be related to several factors. Most notably amongst these is that manufacturing times were significantly longer than in other similar studies (median of 52 days) which led to delays for patients and development of progressive disease which was an end point of the study. Furthermore, the pattern of use of bridging therapy where some received more than one cycle may also have played a role. Additional differences in prognostic characteristics between the two arms and choices of therapy allowed in the standard of care arm likely further contributed to the lack of response noted.

Several studies are ongoing evaluating tisa-cel for other indications. A phase 3 study (NCT 05888493) is ongoing evaluating tisa-cel compared to standard of care chemoimmunotherapy in patients with R/R FL. A phase two study is ongoing evaluating tisa-cel in high-risk pediatric and young adult patients with B-ALL who are minimal residual disease (MRD) positive at the end of consolidation treatment.

## Brexucabtagene autoleucel

Brexucabtagene autoleucel (brexu-cel, Tecartus©) is a CD19 CAR product. It is manufactured using a gamma retroviral vector followed by appropriate processing, and uses the CD28 costimulatory domain. It is identical to axi-cel in antigen binding domain but different because the manufacturing process included an additional sorting step to exclude leukemia cells before CART transduction. This difference in manufacturing process is why it is a different product.

Brexu-cel was evaluated for use in patients with R/R mantle cell lymphoma (MCL) in the ZUMA-2 study (NCT02601313).^9^ The majority of patients included were refractory to three or more prior lines of therapy, and all had received a prior Bruton Tyrosine Kinase inhibitor (BTKi). In this study the estimated 12-month OS was 83% and brexu-cel received an accelerated FDA approval based on these results pending confirmatory studies. Brexu-cel was also evaluated in B-ALL in the ZUMA-3 study (NCT02614066).^10^ In this study which included adult patients with R/R disease, median OS was 18.2 months, leading to FDA approval for this indication.

Ongoing studies evaluating brexu-cel include ZUMA-25 (NCT 05537766) which is evaluating brexu-cel in rare B-cell malignancies including R/R Waldenstrom macroglobulinemia, R/R Richter transformation, R/R Burkitt lymphoma, and R/R hairy cell leukemia. The ongoing ZUMA-4 study (NCT 02625480) is evaluating brexu-cel in pediatric and adolescent patients with R/R B-ALL or R/R LBCL.

## Lisocabtagene maraleucel

Lisocabtagene maraleucel (liso-cel, Breyanzi©) is a CD19 CAR product that is manufactured using a lentiviral vector. It contains the 4-1BB costimulatory domain but also includes a non-functional epidermal growth factor receptor (EGFR) protein that is co-expressed on the cell surface.

Liso-cel was evaluated in the TRANSCEND NHL 001 study (NCT02631044) which included patients with R/R LBCL including DLBCL, transformed indolent lymphoma, HGBCL, PMBCL, and FL grade 3B.^11^ This study demonstrated a RR of 73% in a heavily pretreated population which lead to FDA approval for R/R LBCL. The subsequent TRANSFORM study (NCT02631044) compared treatment with liso-cel to ASCT in patients with LBCL whose disease was primary refractory or had relapsed within 12 months of first line therapy.^12^ Median event free survival in the liso-cel group was 10.1 months compared to 2.3 months in the ASCT arm, and these findings lead to approval of liso-cel in the second line for this patient population.

Ongoing studies are evaluating liso-cel in combination with various targeted agents for the treatment of Richter transformation and R/R LBCL (NCT05672173 and NCT05873712).

## Idecabtagene vicleucel

Two CAR T-cell products have been approved for use in multiple myeloma (MM), both targeting the B-cell maturation antigen (BCMA). The first of these, idecabtagene vicleucel (ide-cel, Abecma©) is manufactured with a 4-1BB costimulatory domain and was studied in the KarMMa trial (NCT03361748).^13^ This trial included adult patients with R/R MM with disease refractory to at least four prior lines of therapy including an immunomodulatory agent, a proteasome inhibitor, and a CD38 monoclonal antibody as part of the four or more lines. In this study RR was 73% and median progression free survival (PFS) was 8.8 months. Based on these results ide-cel received FDA approval in 2021. In the subsequent KarMMa-3 trial (NCT03651128), ide-cel was compared with standard of care therapy in patients with R/R MM who had received between 2 and 4 prior regimens.^14^ Patients in the ide-cel arm had superior RR (71% compared to 42% in the standard of care group) and improved median PFS of 13.3 months compared to 4.4 months. The FDA is currently reviewing a supplemental application for this label indication.

Ongoing studies evaluating ide-cel include KarMMa-2 (NCT 03601078) which is evaluating ide-cel in patients with R/R MM and high-risk MM. KarMMa-9 (NCT 06045806) is evaluating the use of ide-cel followed by lenalidomide maintenance versus lenalidomide maintenance alone in patients with newly diagnosed MM who have a suboptimal response to autologous stem cell transplantation.

## Ciltacabtagene autoleucel

Ciltacabtagene autoleucel (cilta-cel, Carvykti©) is also manufactured from autologous lymphocytes and contains a 4-1BB costimulatory domain. Cilta-cel was evaluated in the CARTITUDE-1 trial (NCT03548207) in patients with R/R MM with disease refractory to at least four prior lines of therapy including an immunomodulatory agent, a proteasome inhibitor, and a CD38 monoclonal antibody as part of the four or more lines.^15^ In this study ORR was 97% and 12-month OS was 89%, which lead to FDA approval of cilta-cel in this population in 2022. The subsequent CARTITUDE-4 study (NCT04181827) evaluated cilta-cel compared to standard of care in the second line in patients who had received between 1 and 3 prior lines of therapy and were considered lenalidomide-refractory.^16^ 12-month PFS in the cilta-cel arm was 75.9% compared with the standard of care arm, and the cilta-cel arm also had improvements in ORR and complete RR. Based on this, FDA review is currently pending for cilta-cel as second-line therapy.

A phase 2 study is ongoing evaluating the safety and efficacy of cilta-cel in patients with high-risk smoldering MM. The CARTITUDE-5 study (NCT04923893) is evaluating non-daratumumab based induction followed by either cilta-cel or maintenance therapy in patients with newly diagnosed MM who are not being considered for autologous stem cell transplant. The CARTITUDE-6 (NCT05257083) study is evaluating daratumumab based induction followed by cilta-cel compared with daratumumab based induction followed by autologous stem cell transplant in newly diagnosed MM.

### CART Design Considerations

The existing FDA approved CART products in 2023 are second generation CARs based on whether they use CD28 or 4-1BB costimulatory signaling capability. CARs that possess CD28 costimulatory agent proliferate very rapidly, have a shorter time to peak level, and are associated with higher grades of CRS and other toxicities. CARs with 4-1BB also have great proliferation capacity but not as rapidly as CD28. Furthermore, CARs with 4-1BB are associated with less toxicity and persist much longer than CD28. Interestingly, the only CAR that has demonstrated overall survival in a randomized setting is a CD28 CAR.^3^

## Clinical Utility of CAR T

### Large B-cell Lymphoma

Several CAR T products are available for the treatment of LBCL, which underscores the importance of appropriate patient and product selection. For second-line treatment in patients who are primary refractory or who have developed relapsed disease within twelve months of primary treatment both axi-cel and liso-cel can be considered. When choosing between the two products it is notable that liso-cel had slightly lower rates of severe CRS and ICANS reported but a longer manufacturing time than axi-cel, with the important caveat that cross-trial comparisons should be limited. Given this, it may be reasonable to treat younger, more fit patients with axi-cel and to consider liso-cel for those who are older or have more comorbidities or worse performance status.

For third line and beyond, axi-cel, tisa-cel, and liso-cel are all options. This again underscores the importance of taking patient related factors into account. Specifically, tisa-cel or liso-cel may be more appropriate treatments for older, less-fit patients compared to axi-cel based on the toxicity rates noted in the various studies referenced above.

In both LBCL and FL when choosing between products it is also important to consider manufacturing times and potential delays in treatment when matching patients with CAR T products. Patients with more aggressive or rapidly proliferative disease may require products that can be manufactured more quickly or risk requiring additional lines of bridging therapy, which can contribute to worsening performance status and add to cost of overall treatment.

### Follicular Lymphoma

Both axi-cel and tisa-cel are options for patients with R/R FL. Again, when choosing between these two products axi-cel may be more appropriate for patients who are younger and with good performance status, while tisa-cel may be more appropriate for older, more frail patients.

### Multiple Myeloma

Both ide-cel and cilta-cel are approved for treatment of patients with heavily pretreated R/R MM. Additionally, both products are currently under review for approval as earlier lines of therapy for patients with R/R disease. In the study that led to its approval cilta-cel was found to have higher rates of grade 3 or 4 ICANS, which may limit its use in an older, more frail patient population or in patients with pre-existing comorbidities.

### Other

Finally, there are case reports and small retrospective series of patients who have been treated off-label with CD19-targeted CAR T-cells for secondary central nervous system (CNS) lymphoma, post-transplant lymphoproliferative disorder (PTLD), chronic lymphocytic leukemia (CLL) with Richter’s transformation, and HIV-associated lymphomas.^17–20^ These reports largely show some efficacy of CAR T treatment in these populations, though reports are limited by small numbers of patients and warrant larger studies before recommendations can be made about use of axi-cel for these patients.

### Long term CART limitations

Though highly effective and have led to prolongation of life in hematological malignancies, CARTs are associated with variable degree of persistent cycopenias lasting for more than 3 months in 20 – 40% of patients, and recently, the FDA has issued a warning about T cell malignancies in recipients of BCMA- or CD19-directed autologous CAR T cell immunotherapies.^21,22^

## Conclusion

Multiple CAR T-cell therapy products have been approved for use across several different malignancies including LBCL, B-ALL, and MM. The development of CAR T-cells has dramatically changed treatment for several hematologic malignancies and, with longer follow up, may prove to be curative for a subset of patients. For many patients who undergo CAR T treatment, this therapy provides meaningful long-term remissions and, most importantly, allows them to enjoy a good quality of life.

Though dramatic toxicities have been noted in treatment patients with CAR T-cells remarkable advances have been made in management of these toxicities including the use of steroids both as treatment and as prophylaxis, and the incorporation of early tocilizumab use into toxicity management protocols. Further investigation is ongoing into longer-term or less frequently encountered toxicities such as hypogammaglobulinemia, prolonged cytopenias, and CAR T-associated hemophagocytic lymphohistiocytosis (HLH). These studies will only serve to make CAR T therapy safer and more accessible for patients in the future.

Several CAR T products have been approved for use in the second line, and studies are ongoing to advance them further in the treatment paradigm, most notably in LBCL. As these studies proceed it will be important to continue to identify optimal patients to be treated with each individual product as well as to identify the optimal timing for using CAR T therapy. Additionally, choosing patients appropriately, learning more about the best use of bridging therapy, optimizing cost and insurance approval processes, and learning more about toxicity management will continue to make CAR T therapy safer and more accessible for patients.
